# Comparing COI and ITS as DNA Barcode Markers for Mushrooms and Allies (*Agaricomycotina*)

**DOI:** 10.1371/journal.pone.0025081

**Published:** 2011-09-22

**Authors:** Bryn T. M. Dentinger, Maryna Y. Didukh, Jean-Marc Moncalvo

**Affiliations:** 1 Department of Ecology and Evolutionary Biology, University of Toronto, Toronto, Ontario, Canada; 2 Department of Natural History, Royal Ontario Museum, Toronto, Ontario, Canada; 3 Jodrell Laboratory, Royal Botanic Gardens, Kew, Richmond, Surrey, United Kingdom; University of Veterinary Medicine Hanover, Germany

## Abstract

DNA barcoding is an approach to rapidly identify species using short, standard genetic markers. The mitochondrial cytochrome oxidase I gene (COI) has been proposed as the universal barcode locus, but its utility for barcoding in mushrooms (ca. 20,000 species) has not been established. We succeeded in generating 167 partial COI sequences (∼450 bp) representing ∼100 morphospecies from ∼650 collections of *Agaricomycotina* using several sets of new primers. Large introns (∼1500 bp) at variable locations were detected in ∼5% of the sequences we obtained. We suspect that widespread presence of large introns is responsible for our low PCR success (∼30%) with this locus. We also sequenced the nuclear internal transcribed spacer rDNA regions (ITS) to compare with COI. Among the small proportion of taxa for which COI could be sequenced, COI and ITS perform similarly as a barcode. However, in a densely sampled set of closely related taxa, COI was less divergent than ITS and failed to distinguish all terminal clades. Given our results and the wealth of ITS data already available in public databases, we recommend that COI be abandoned in favor of ITS as the primary DNA barcode locus in mushrooms.

## Introduction

DNA barcoding is an approach to rapidly identify species using short, standard genetic markers [1], [2], [3], [4], [5]. The DNA barcoding approach to documenting diversity is particularly useful for groups of cryptic organisms like bacteria and fungi. Indeed, the challenge of documenting the 712 K to >15 million species of fungi, 90–95% of which remain undescribed [5], [6], [7], [8], [9], [10], is more tractable with molecular than with traditional methods, which add only about 1000 new species a year [11]. However, until recently, there has been very little effort to standardize the methods for molecular identification of fungal species and no one marker has been formally selected as a DNA barcode region in fungi.

To qualify as a DNA barcode region, a locus should be easy to amplify from most or all species in the target group using universal primers and show low intra-specific and high inter-specific divergence (creating a “barcode gap”). For animals, the mitochondrial cytochrome oxidase I (COI) locus appears to satisfy these criteria for most groups [1], [12], [13], [14]. However, COI has not been shown to be very effective outside of animals, although studies are limited. The plant barcoding initiative (http://www.barcoding.si.edu) has identified several alternative loci that may be used together for a multilocus barcode for land plants [15], [16], [17], [18], [19]. In fungi, the nuclear internal transcribed spacers of the ribosomal repeats (ITS), and less so divergent domains D1–D2 of the largest subunit of the ribosomal RNA (LSU), have long been used for this purpose [5], but length variation in these regions make sequence alignment difficult across divergent taxa, and there is still a lack of procedure standardization. COI may provide an advantage over these loci because alignment of this locus across a divergent set of taxa is trivial.

Although the idea of DNA barcoding is both essential to and already an informal part of much current research with fungi, only a few studies exist that examined the effectiveness of COI as a DNA barcode [20], [21], [22], [23], [24]. In the *Ascomycota*, COI was shown to be more effective than ITS, but less than beta tubulin A, for distinguishing among species of *Penicillium* subgenus *Penicillium* [21] while in *Neohumicola* COI and ITS provided similar resolution [22]. In contrast, Geiser et al. [20] and Gilmore et al. [23] reported low divergence between COI homologs in *Aspergillus* and *Fusarium,* respectively, and more critically, detected COI paralogs. In the *Basidiomycota*, Vialle et al. [24] were unable to amplify and sequence COI from most of the rust fungi (*Urediniomycetes*) they examined due to the presence of introns in the priming and sequencing regions and, when they did obtain sequences, variation was inferior to ITS and LSU. At least four introns occur in the COI coding region of the mushroom *Agrocybe aegerita* [25], 15 in *Trametes cingulata* [26], 19 in *Agaricus bisporus* [27], and either one (*Agrocybe aegerita*), two (*Crinipellis perniciosa*), or eight (*A. bisporus*) introns have been reported from the 600 bp “barcoding region” at the 5′-end of the gene [21], [27], suggesting that introns may also occur in other mushrooms and could be problematic as in the rust fungi. Overall, the utility of COI as a DNA barcode for species-level identification of mushrooms remains to be investigated comprehensively.

Here, we set out to facilitate the formalization of a DNA barcoding effort in mushrooms and their relatives (*Basidiomycota*:*Agaricomycotina*), representing about one-third of all known species of fungi [∼20,000 described species; 11], by evaluating COI as a DNA barcode for the group. The criteria we used included the ability to design universal primers, amplification success, and capacity to diagnose species using a phylogenetic approach. We also compared COI with ITS, the latter being an informal “standard” marker for species resolution in *Agaricomycotina*.

## Materials and Methods

### Ethics Statement

Newly collected specimens used in this study were obtained with permission from public and private lands. The permit for collecting specimens in Catoctin Mountain National Park was provided by the United States National Park Service to Steven Stephenson (CATO-2006-SCI-0005) and the permit for collecting specimens in Algonquin Provincial Park was provided by Ontario Parks to Jean-Marc Moncalvo (s.n.).

### Taxon Sampling

Approximately six hundred and fifty mushrooms in three classes of *Agaricomycotina* (*Tremellomycetes*, *Dacrymycetes*, *Agaricomycetes*) were newly collected from Ontario, Québec, and Maryland for this study. Species identifications were made comparing macro-and micro-morphological features with species descriptions in the relevant taxonomic literature (too numerous to list). Vouchers of all newly collected specimens are deposited in the fungal herbarium of the Royal Ontario Museum (TRTC). Additional specimens used in this study were borrowed from other herbaria (MIN, HSC, BUF, LIP, SFSU, WTU, KUN, TMI, UC, USJ, and NY).

### DNA extraction and sequencing

**Figure 1:**
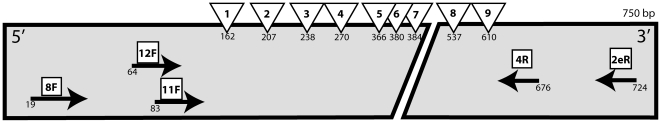
Positions of the four most successful PCR primers and introns encountered in mushroom cytochrome oxidase I (COI) genes relative to COI exon regions of *Agrocybe aegerita* (GenBank Accession AF010257).

DNA was extracted following various standard protocols [28], [29] or using a new rapid DNA isolation protocol developed in our lab [30]. Approximately 530-666 bp of exon from the 5′ end of the COI gene was amplified by designing taxonomically nonspecific primers (Table 1 and Figure 1) based on an alignment of fungal COI sequences in GenBank (*Cryptococcus neoformans* AY560609, *Moniliophthora perniciosa* NC005927, *Schizophyllum commune* AF402141, *Agrocybe aegerita* AF010257). PCR amplification was achieved using a cycling program with an initial denaturation of 95C for 2 min, followed by 30 cycles of denaturation at 94 C for 45 sec, annealing at 60 C for 45 sec, and extension at 72 C for 1 min 10 sec, followed by a final extension at 72 C for 7 min and an indefinite refrigeration at 4 C. New primers “COXBOL1-F” and “COXBOL1-R” were also designed for improved amplification of COI from genomic DNA for *Boletales* (Table 1) based on an initial alignment of *Boletales* sequences obtained using general primers. A touchdown program was used with the *Boletales*-specific primers: initial denaturation step for 2 min at 94 C, followed by 5 cycles of 94 C (30 sec), 55 C (30 sec), and 72 C (1 min), followed by 25 cycles of 94 C (30 sec), 50 C (30 sec), and 72 C (1 min), and ending with a final extension step at 72 C for 7 min. The ITS region was amplified and sequenced with primers ITS1F and ITS4 using standard protocols [31], [32] or with primers ITS8F and ITS6R using a new high-throughput protocol [30]. PCR products were visualized by UV fluorescence after running out 3-25 µL PCR products in a 1% agarose gel containing 0.005% ethidium bromide. Prior to sequencing, positive PCRs were cleaned one of two ways: 1) by incubating samples for 15 min at 37C then 15 min at 80 C after adding 0.4 volumes of a mixture containing shrimp alkaline phosphatase (0.05 units/µL) and exonuclease I (0.05 units/µL) in water, 2) loading the entire PCR in a single lane, running the gel at 90 V for ca. 1 h, cutting the band from the gel using a clean razor, placing the excised gel in the top of a disposable pipette tip with a filter and trimmed to ∼1 cm in length, placing the pipette tip in a 1.5 mL microfuge tube, and collecting the liquid in the gel by spinning the tube for 10 min at 10,000 g. Unidirectional dye-terminator sequencing used the ABI BigDye kit (Foster City, CA) and reactions were run on an ABI PRISM 3100 DNA Analyzer in the Department of Natural History at the Royal Ontario Museum, Toronto, after ethanol precipitation and resuspension in HiDi formamide. Sequences were edited using Sequencher3.1 (GeneCodes, Ann Arbor, MI) and are available as a project on the Barcode of Life Data Systems online database [“Evaluating COI in mushrooms and allies (Agaricomycotina)” Project code YYY; www.barcodinglife.org; 33] and through GenBank [Accession numbers JN020964-JN021114 (ITS), JN029360-JN029526 (COI); 34]. Additional sequences obtained from GenBank that were used in this study include the following accession numbers: EU231946, EU231948, EU231949, EU231954, EU231958, EU231966, EU231969, EU231971, EU231980, EU231982, EU231983, EU231984, EU231985, EU231990, EU231991, EU231992.

**Table 1:** Primers designed for amplification of COI from *Agaricomycotina*.

Primer name	Primer sequence (5′→3′)	Primer direction	Starting position relative to *A. aegerita*
5F	TGRTTAAATTCHACHAAYGC	forward	7
8F	ACHAAYGCWAARGANATWGG	forward	19
Ag-3F	AGGTACCCTTTATTTAATTTTTGCT	forward	36
6F	GGWACMCTDTATYTDATNTTTGC	forward	37
9F	GGAACGCTGTACTTAATTTTTGC	forward	37
12F	TTYKCDGGDATGATHGGDACDGC	forward	64
11F	GGDATGATHGGDACDGCHTT	forward	70
4F	ATHGGWACWGCYTTYTCHG	forward	76
COXBOL1-F	GACGGCATTTTCWGTTCTTATTAG	forward	81
10F	AGGAACGCTGTACTTSSTTTTTGC	forward	83
13F	AAYGTWATAATWWCWGCTCATGC	forward	160
COXBOL1-R	GATCATARAAACTWGTATTAAAGTTC	reverse	661
4R	CWCCWCCWCCAGCWGGRTC	reverse	676
5R	GTTGATAWARWATWGGRTC	reverse	694
2eR	CYTCNGGRTGACCRAARAAYC	reverse	724
7R	GCVGCWGTRGARTARGCTCTHGWA	reverse	916
6R	GCNGCWGTYAAYTANGCRC	reverse	917

### Alignment and Phylogenetic Analyses

#### COI

Intron sequences from COI were removed before aligning the exon regions using ClustalW2 [34] as implemented in SeaView v4.2.9 [36], [37]. The best-fitting evolutionary model for the data was selected using the AIC in jModelTest v0.1.1 [38]. Phylogenetic analysis under the maximum likelihood framework was conducted using PhyML 3.0 [39] as implemented in SeaView v4.2.9 from 10 random starting trees and selecting the best of NNI and SPR branch swapping tree searching strategies. Branch support was estimated using the approximate likelihood ratio test (aLRT) function [40] implemented in PhyML 3.0. Intra- and inter-specific divergences were calculated using the Kimura two-parameter model (K2P)-corrected distances using the x86 version of PAUP*v4.0d90 [41].

#### ITS

Complete ITS sequences were trimmed to begin and end with the conserved motifs 5′-(...gat)CATTA— and —GACCT(caaa...)-3′ to facilitate alignment. These motifs correspond to the conserved 3′ and 5′ termini of the flanking SSU and LSU genes, respectively. Because ITS sequences are length-variable regions with rapid substitution rates, a global alignment cannot be unambiguously constructed. Therefore, in order to ensure proper assessment of character homology between sequences, we identified “alignment groups” of sequences with 80% or greater similarity, a threshold that enabled unambiguous alignment. Any sequences that were greater than 20% dissimilar to each other were assumed to represent different species (intraspecific ITS variability in fungi was calculated to be 2.51% with a standard deviation of 4.57 [42]). To quickly identify these alignment groups, we utilized the “contig assembly” algorithm in Sequencher 4.10.1 (GeneCodes, Ann Arbor, MI). We first identified (based on annotated GenBank records) and removed the 5.8S subunit that separates the ITS1 and ITS2 regions to eliminate problems associated with high homology in this highly conserved ribosomal region but low homology in the flanking ITS regions. Second, we imported a FASTA file containing the concatenated sequences of ITS regions 1 and 2 sequences into Sequencher. Next, we generated alignment groups using the “Assemble Automatically” procedure with “Minimum Match Percentage” set to 80, “Minimum Overlap” set to 100 bp, and the contig consensus type set to “Consensus Inclusively.” The sequences in the “contig” folders were then exported and aligned using the default options in MUSCLE v3.8.31 [43] as implemented in SeaView v4.2.9. Of these aligned files, those with 3 taxa were clustered in PAUP*4.0b10 based on K2P-corrected distances using the BioNJ algorithm and the “Break Ties” option set to random. For all alignments with >3 taxa, maximum likelihood searches under the GTR+G+I model were conducted using SeaView as described above. Intra- and inter-specific divergences were calculated using the Kimura two-parameter model (K2P)-corrected distances in PAUP*.

For direct comparison of intra- and inter-specific divergences, genetic distances were calculated in PAUP*v4b10 [41] using pairwise distances corrected using the Kimura 2-Parameter (K2P) model for all sequences within the ITS alignment groups and their corresponding COI sequences. For each dataset, the mean, median, and maximum intra-specific distances and the mean, median, and minimum inter-specific distances were determined. Inter-specific distances were calculated by comparing the minimum distance between each species and its sister taxon. Ratios of minimum inter- to maximum intra-specific distances were plotted in Excel (Microsoft, Seattle, WA).

## Results

### Taxon Sampling

Our sampling included ca. 184 species in 73 genera (as identified by morphological traits). More than 90% of specimens (144 spp. in 49 genera) are members of the principal gilled-mushroom subclass *Agaricomycetidae*. Of the remaining, ca. 40 species belong to the *Tremellomycetes*, *Dacrymycetes*, and other orders of *Agaricomycetes* excluding the subclass *Phallomycetidae* [44].

### DNA extraction and sequencing

Only 204 specimens (31.4%) yielded COI sequences, representing 57.7% and 61.3% of all species and genera sampled, respectively. The most successful primer combinations for COI amplification were 11F/2eR (615 bp amplicon), 12F/4R (529 bp amplicon), and 8F/2eR (666 bp amplicon). Most COI sequences used for this study were generated using combinations 11F/2eR or 12F/4R and trimmed to approximately 450 bp. Introns were found in 14 sequences (∼7% of those that amplified) in 18 species from 12 genera. Introns occurred in nine different locations (Fig. 1). Identical insertion/deletion sites occurred in sequences from specimens of different genera (e.g., *Marasmius androsaceus*, and *Lepiota clypeolaria* have an intron inserted at position 4), but these locations were not conserved within genera (e.g., *Laccaria*). In addition, presence/absence of intron was not always consistent between multiple specimens of the same species (e.g., *Amanita fulva*).

### Alignment and Phylogenetic Analyses

A total of 167 specimens from 102 morphospecies of *Agaricomycetidae*, including 97 taxa belonging to the *Boletales* and *Agaricales* and 5 taxa belonging to the *Polyporales* (*Ganoderma*, *Merulius*, *Polyporus*), *Russulales* (*Albatrellus*), and *Thelephorales* (*Sarcodon*), were selected to compare COI with ITS. Thirty-six of the 102 morphospecies were represented by more than one sequence (2–9 sequences/species). Phylogenetic analysis of the COI dataset resulted in a moderately supported tree (aLRT≥0.95) and generally reflects the known topology of the *Agaricomycetidae*, except for the placement of *Polyporus badius* near *Suillus* and a non-monophyletic *Boletales*.

Intra- and inter-specific divergence was calculated for 30 species represented by more than one sequence in both the COI and ITS datasets based on global alignments of each gene (Table 2). For COI, the mean±standard error intra-specific divergence was 0.0009±0.0004 (0.0000 median, 0.0064 maximum) and mean±standard error inter-specific divergence was 0.0569±0.0079 (0.0428 median, 0.0043 minimum). For ITS, the mean±standard error intra-specific divergence was 0.0063±0.0030 (0.0011 median, 0.0747 maximum). In our calculation, inter-specific divergence for ITS was set at 21% when the divergence was greater than 20% (a minimum estimate of divergence); mean±standard error inter-specific divergence was 0.1499±0.0156 (0.2100 median, 0.0258 minimum).

**Table 2:** Comparison of COI and ITS divergences using maximum intra-specific and minimum inter-specific divergences for the set of ITS alignment groups identified using Sequencher.

Dataset	COI (intra-/inter-)	ITS (intra-/inter-)
*Amanita flavoconia* (n = 2)	**0.0000/0.0930**	0.0022/0.0511
*Amanita porphyria* (n = 2)	0.0000/0.1275	**0.0000/—** 1
*Boletus badius* (n = 2)	0.0000/0.0439	**0.0000/—**
*Boletus edulis* (n = 9)	*0.0062/0.0020*	**0.0104/0.0497**
*Boletus nobilissimus* (n = 4)	0.0000/0.0020	**0.0039/0.0223**
*Boletus regineus* (n = 4)	*0.0041/0.0020*	***0.0142/0.0096***
*Boletus rex-veris* (n = 2)	*0.0020/0.0020*	**0.0029/0.0308**
*Boletus variipes* (n = 2)	**0.0000/0.0145**	0.0048/0.0538
*Catathelasma ventricosa* (n = 2)	0.0000/0.1055	**0.0043/—**
*Clitocybe* sp. (143Alg, 38Alg) (n = 2)	0.0000/0.0860	**0.0091/—**
*Collybia cirrhata* (n = 3)	**0.0000/0.0129**	0.0000/0.0345
*Entoloma clypeatum* (n = 2)	0.0000/0.1276	**0.0000/—**
*Entoloma sinuatum* (n = 2)	0.0000/0.0762	**0.0028/—**
*Entoloma* sp. (parasite on *Tricholoma focale*) (n = 2)	0.0064/0.0417	**0.0000/0.0390**
*Hygrocybe conica* (n = 3)	0.0000/0.0260	**0.0329/—**
*Hygrocybe lacmus* (n = 2)	0.0000/0.1201	**0.0083/—**
*Hygrocybe miniata* (n = 2)*	0.0064/0.0238	**0.0747/—**
*Hygrophorus agathiosmus* (n = 2)	0.0043/0.0373	**0.0000/—**
*Hygrophorus flavodiscus* (n = 3)	0.0000/0.0283	**0.0025/—**
*Hygrophorus pudorinus* (n = 3)	0.0000/0.0554	**0.0056/—**
*“Inferiboletus”* (n = 2)	0.0000/0.0335	**0.0015/—**
*Laccaria* spp. (n = 2)	N.A./0.0107	N.A./0.0575
*Lacrymaria*/*Psathyrella* (n = 2)	N.A./0.0394	N.A./0.0837
*Leccinum vulpinum* (n = 2)	0.0000/0.0208	**0.0050** 2 **/—**
*Lepiota castanea*/*Lepiota* sp. 16JS06 (n = 2)	N.A. /0.0305	N.A./0.0313
*Pholiota* sp. (n = 3)	0.0000/0.1051	**0.0000/—**
*Pluteus* sp.A (n = 4)	**0.0021/0.0216**	0.0000/0.0733
*Pluteus* sp.B (n = 2)	0.0000/0.0043	**0.0000/0.0323**
*Pluteus* sp.C (n = 2)	**0.0021/0.0043**	0.0186/0.0258
*Pluteus* sp.D (n = 2)	0.0000/0.0173	**0.0000/0.0258**
*Pluteus* sp.E (n = 2)	**0.0000/0.0173**	0.0067/0.0390
*Psathyrella* 72CAT06/151CAT06 (n = 2)	N.A./0.0693	N.A./0.1207^3^
*Psathyrella gracilis* (n = 2)	0.0000/0.0786	**0.0022/—**
*Stropharia ambigua* (n = 4)	0.0000/0.1432	**0.0000/—**
*Suillus cavipes* (n = 2)	0.0000/0.0328	**0.0000/0.0662**
*Tricholoma inamoenum* (n = 3)	0.0043/0.0260	**0.0000/—**
*Tricholoma sejunctum* group (n = 3)	N.A./0.0107	N.A./0.1136
*Xerocomus subtomentosus* (n = 2)	0.0000/0.0271	**0.0022/—**

Distances in italicized text indicate intraspecific distances greater than or equal to the interspecific distance. Distances in bold text indicate the least intra-:inter- specific distance ratio.

1 “—” indicates inter-specific distance is >20%.

2 incomplete sequence present; only first 408bp included.

3 incomplete sequence present; first 213 bp excluded.

*may be two cryptic species.

A side-by-side comparison reveals that COI and ITS distances are qualitatively similar (Table 2). Except for three cases in *Boletus* spp., all ratios of inter- to intra-specific divergences for COI and ITS were greater than one, the minimum threshold for a barcode locus (Table 2). In general, intra-specific divergences were slightly higher for ITS than COI. Inter-specific divergence was also higher for ITS than COI except between *Amanita flavoconia* and *A. rubescens*, where minimum inter-specific divergence was great for COI than ITS. The highest maximum intra-specific divergence was 7.47% for ITS (*Hygrocybe miniata*) and 0.64% for COI (*Entoloma* sp. parasite on *T. focale* and *Hygrocybe miniata*). The lowest minimum inter-specific divergence for ITS was 0.96% (*Boletus regineus*) and 0.20% for COI (*Boletus* spp.).

**Figure 2:**
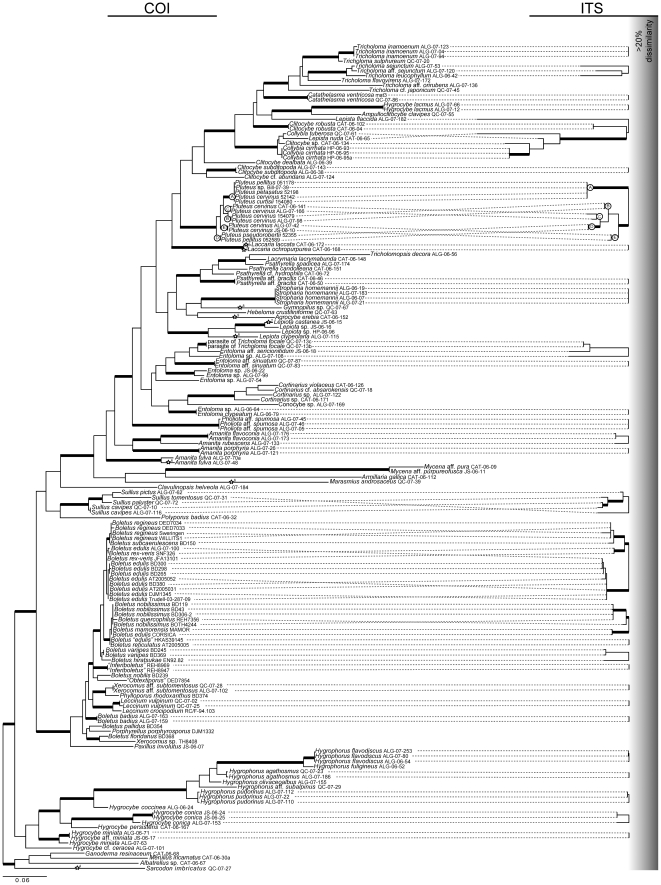
PhyML phylogram of 167 partial COI (left) and PhyML or Neighbor-joining distance phylograms of full and partial ITS (right) sequences from ∼100 species of mushrooms. Thickened lines represent branches that received ≥0.95 aLRT. Stars indicate COI sequences with an intron in the barcode region. Superscript numbers next to stars correspond to the position of the intron (Fig. 1). Branching for ITS sequences reflect ML- or NJ-based analysis of contigs formed using Sequencher; branching is absent where inter-specific distances are greater than the 20% cutoff value used in the Sequencher contig assembly. Letters at nodes in the genus *Pluteus* represent the phylogenetic species (reciprocally monophyletic with posterior probability of ≥0.95) of the *Pluteus cervinus* complex.

COI and ITS largely agree on terminal taxa, which corresponds well with morphospecies (Fig. 2). Discrepancy between terminal taxa recovered by COI and ITS was limited to the porcini group of *Boletus* where intraspecific sampling was most extensive. In one case, ITS recovers *B. nobilissimus* as distinct from *B. quercophilus*, but COI does not. Similarly, ITS recovers a monophyletic *B. edulis* sensu stricto [45], whereas COI groups *B. rex-veris* with one specimen of *B. edulis*.

## Discussion

### Amplification of COI

Like Vialle et al. [24] in a different group of basidiomycetes (rusts), we encountered low PCR success with COI in mushrooms. Even using multiple combinations of primers, our success rate was poor. Post-PCR processing of COI resulted in even worse success rates because multiple, large introns made sequencing difficult. In our set of sequences, COI exons may be interrupted by one or more large introns (∼1500 bp) with variable positions that are not phylogenetically conserved (Fig. 1, 2). Thus, even after considerable effort we succeeded in generating COI sequences for only ∼30% of the specimens, a rate much lower than typically observed for ITS [e.g, 30]. We also encountered possible paralogous copies of COI from some specimens that were sequenced twice or more (data not shown), confirming in mushrooms similar problems recently reported with this locus for *Fusarium* [23]. Moreover, unlike the ITS, designing universal COI primers for mushrooms (let alone all fungi) is not possible. Thus, each order, family, or even genus would probably require extensive troubleshooting and primer optimization, which is time-consuming, costly, antithetical to high-throughput data acquisition, and impractical for detection of fungi from environmental samples.

### Performance of COI versus ITS as barcodes

Overall, the performances of COI and ITS as barcode markers for mushrooms are similar. Both generally exhibit low intra-specific and high inter-specific divergences in our dataset and both were able to distinguish most of the morphospecies we sampled (Table 2; Fig. 2). However, COI failed to recover two morphospecies (*Boletus edulis* and *B. nobilissimus*) that are recovered as reciprocally monophyletic by ITS, in agreement with a recent multigene phylogeny [45]. Moreover, the ratio of maximum intraspecific to minimum interspecific distance was greater for ITS than COI in 27/33 comparisons (82%; Table 2), indicating that ITS has a more pronounced “gap” between species. Amplification and sequencing notwithstanding, ITS appears to be better than COI as a primary DNA barcode locus for mushrooms.

### Alignability of ITS

The inability to produce a reliable alignment of ITS sequences across a divergent set of taxa is a major disadvantage of using ITS as a barcode. We avoided relying on a global alignment of ITS sequences by first identifying “alignment groups” where assessment of homologous characters for a set of sequences is trivial using widely used alignment software. Our approach relied on the contig assembly algorithm employed by Sequencher, which is normally used to assemble complementary sequence trace files output from ABI sequencers. By eliminating the ∼120 bp of the largely invariable 5.8S RNA subunit, increasing the minimum overlap to the maximum (100 bp), and using a threshold of 80% similarity, we avoided the problem of underestimating sequence divergence among a set of sequences, thereby minimizing ambiguous homology assessment. However, there are two related problems with this method: 1) contig folders must contain sets of sequences with 80% or better similarity, but some sequences may be 80% similar to two or more sets of sequences and could legitimately reside in more than one alignment group, and 2) the contig assembly algorithm is likely to be order-dependent (the assembly algorithm in Sequencher is proprietary and therefore unknown), so when a sequence could reside in two folders it may always preferentially be placed in the first contig folder encountered even if its closest relative is in another folder. To explore whether or not the algorithm employed by Sequencher was biasing our contig assembly, we added to the ITS dataset dummy sequences where we modified the number of substitutions (randomly distributed) and gaps (randomly and evenly distributed or at a single site of extension) of the sequence with the greatest inter-specific divergence (*Entoloma* aff. *sericeum* ALG-07-108) from one of the contig folders. Our dummy sequences ranged from 1–20% divergent from the original. Except for two situations, where gaps are randomly distributed across over 15% or more of the sequence and when gaps are evenly distributed across 12% or more of the sequences (an extra base inserted every 8^th^ position), Sequencher always correctly placed the two closest sequences together. Although the two cases where Sequencher did not correctly assign the two closest sequences together is of some concern, these synthetic scenarios are not very likely to be encountered with real biological material and we did not observe any misleading bias using the real data.

Ideally, a global alignment would be possible for phylogenetic-based methods of identification and this is still a characteristic that may be sought in alternative barcoding loci for mushrooms. But the competency of ITS as a barcode demonstrated here and elsewhere, as well as the wealth of ITS data that can be mined from publicly accessible databases such as GenBank and UNITE [46], further recommends it over COI for mushrooms and other fungi [5].

### Conclusion

The barcode locus that was been chosen for animals, COI, will not work for mushrooms, rusts [24], and probably most other fungi [5], primarily due to the variable and unpredictable presence of large introns in the barcode region. Alternatively, the widely used ITS regions work well for identifying mushroom species, as has been determined empirically by mycologists and other fungal researchers for over a decade. Although ITS has limitations [42], [47], there is to date no better single molecular marker for barcoding mushroom species, and its versatility makes it possible to survey and discover new fungi from environment samples. With the development of high-throughput methods for producing ITS barcodes from mushrooms [30] and the relatively low cost of traditional (Sanger) sequencing, coupled with a cross-discipline desire for reliable molecular tools for the identification of environmental samples [48], the foundations have been laid for a worldwide fungal barcoding campaign. It is time to move forward with a global fungal barcoding initiative that adheres to standard protocols, from processing of new field collections to generating barcodes to integrating barcodes with taxonomy.
